# Demodicosis in a Kunekune pig and molecular characterisation of porcine demodectic mites involved: a case report

**DOI:** 10.1186/s13071-023-06101-8

**Published:** 2024-01-24

**Authors:** Lukas Schwarz, Verena Herb, Sophie Dürlinger, Vera Martin, Nina Poláková, Christa Horvath-Ungerböck, Rene Brunthaler, Christian Knecht, Maximiliane Dippel, Jutta Pikalo, Andrea Ladinig, Anja Joachim, Hans-Peter Fuehrer

**Affiliations:** 1 Department for Farm Animals and Veterinary Public Health, University Clinic for Swine, University of Veterinary Medicine Vienna, Vienna, Austria; 2 Clinical Unit of Small Animal Surgery, Ophthalmology, Department for Companion Animals and Horses, University Clinic for Small Animals, University of Veterinary Medicine Vienna, Vienna, Austria; 3 Clinical Unit of Internal Medicine Small Animals, Dermatology, Department for Companion Animals and Horses, University Clinic for Small Animals, University of Veterinary Medicine Vienna, Vienna, Austria; 4 Department of Pathobiology, Institute of Pathology, University of Veterinary Medicine Vienna, Vienna, Austria; 5 Department of Pathobiology, Institute of Parasitology, University of Veterinary Medicine Vienna, Vienna, Austria; 6 Present Address: Fachtierärzte Althangrund, Vienna, Austria

**Keywords:** *Demodex phylloides*, Skin hyperplasia, Isoxazolins, Sarolaner

## Abstract

**Background:**

In January 2021, a female 1-year-old Kunekune was presented at the University Clinic for Swine with severe reduction of the field of vision resulting in prolonged reaction time when targeting barriers, due to moderate to severe thickening of the skin around both orbits also affecting the eyelids.

**Methods:**

Clinical examination revealed skin hyperplasia, nodular enlargement of the skin pores of the axillar and inguinal region. Ophthalmologists decided to remove parts of the thickened periocular skin, followed by histopathological examination.

**Results:**

Once large amounts of demodectic mites were detected by histopathology, demodicosis could be diagnosed and treatment of the pig was started using sarolaner. Morphological and molecular analyses were performed. Histopathological and parasitological exams led to the aetiological diagnosis of demodicosis in the affected Kunekune pig. Severe skin lesions were revealed to be the consequence of an infestation with *Demodex* sp. Morphological analyses confirmed the involvement of *D. phylloides*. Molecular characterization indicated a *Demodex* species closely related to mites documented in wild boar - most probably *D. phylloides* for which no explicit sequences are available in GenBank yet. Treatment with sarolaner (2.6 mg/kg) resulted in a substantial regression of skin lesions, already detectable 1 month after first treatment.

**Conclusions:**

Demodicosis is a very rare disease in pigs that is most probably related to an impaired immune response to the mites. Demodectic mange should be included in the list of differential diagnoses in cases of periocular alterations of the skin of pigs.

**Graphical Abstract:**

**Figure:**
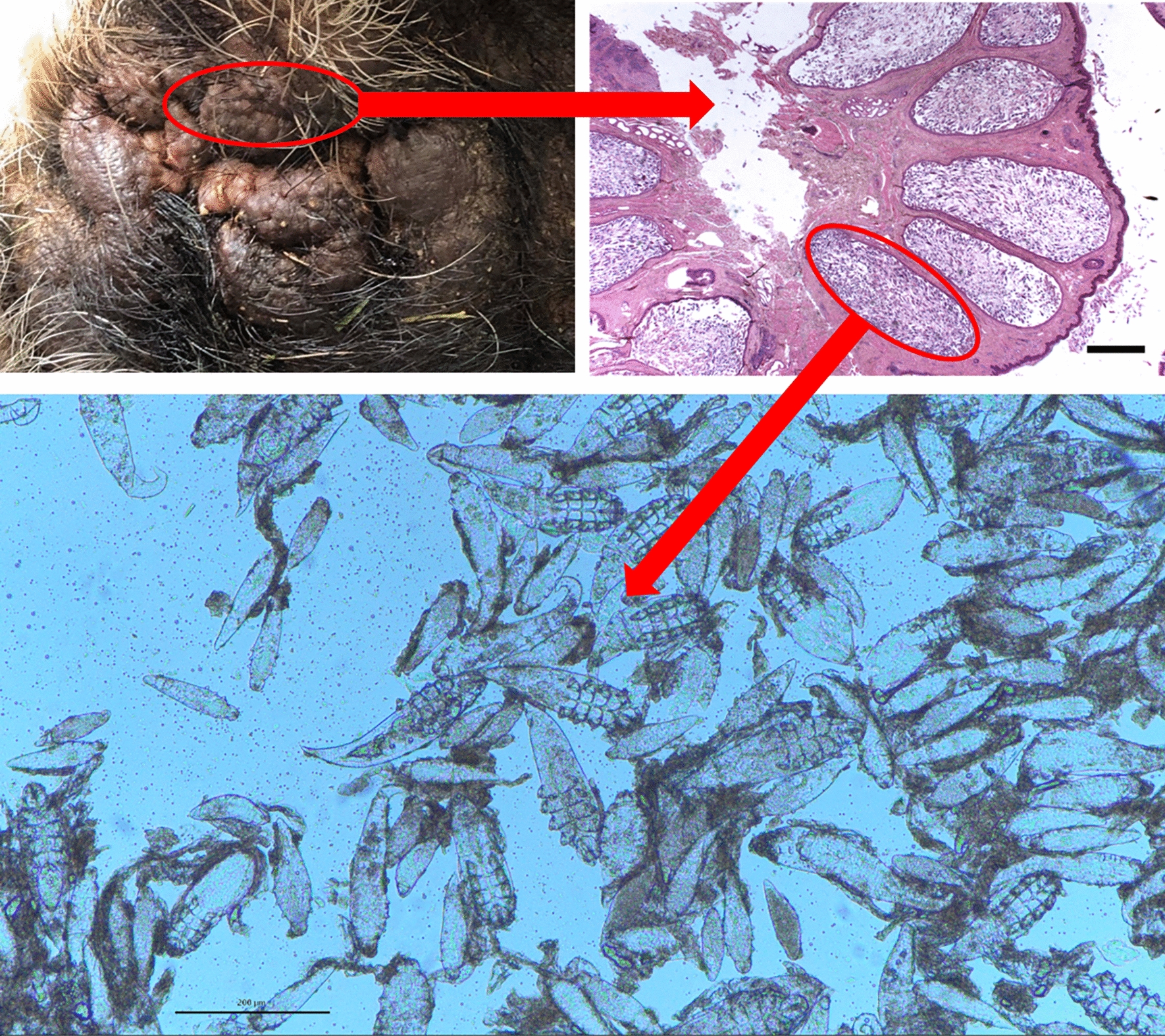

**Supplementary Information:**

The online version contains supplementary material available at 10.1186/s13071-023-06101-8.

## Background

Demodicosis in swine is caused by *Demodex phylloides* [1–3]. The current prevalence of *Demodex* infestation and clinical demodicosis in swine herds is unknown as the infestation is usually perceived as of low relevance for commercial swine production. *Demodex phylloides* usually colonizes the porcine skin and, as clinical demodicosis in pigs is an uncommon condition, the awareness amongst swine veterinarians is poor [4]. *Demodex phylloides* is an elongated mite that reaches a body length of 175–265 µm and lives and reproduces in the hair follicles of pigs [1, 5]. It is transmitted via direct contact [1, 4, 6]. Recent reports of porcine demodicosis are rare [5, 7–9], but historically demodicosis was well known in swine because of the economic impact on leather production [1–3, 6, 10–12]. A study by Fryderyk and Izdebska [9] reported a prevalence of 32% of *Demodex* infestation in wild boars in Poland, though these mites were not found in domestic pigs. Case reports of demodicosis in pigs kept as companion animals have not been described.

The clinical presentation of porcine demodicosis consists of skin-coloured or erythematous papules, plaques, and nodules, which are neither painful nor pruritic and upon squeezing contain thick caseous, whitish material with numerous mites [13, 14]. The skin lesions are localized especially on the snout, eyelids, ventral neck, ventral chest and ventral abdomen as well as medial thighs [13, 14]. Secondary pyoderma can develop, in which case the animals can become pruritic.

In cases with typical clinical presentation, there are few differential diagnoses to consider. Papilloma, parakeratosis secondary to a metabolic or nutritional disorder and pyoderma can be contemplated. If pruritus is present, other parasitic diseases such as sarcoptic mange and pyoderma should be ruled out.

Diagnosis is made by detection of mites belonging to the different developmental stages in deep skin scrapings or in expressed caseous material from skin lesions using light microscopy [2–4], in conjunction with skin lesions [4].

The oral administration of 0.45 g/kg of in-feed ivermectin daily for 7 days is recommended as treatment to reduce mite burden and resolve clinical signs [7, 8]. In case of severely affected commercial pigs, or failure of clinical recovery after treatment, culling might be necessary [4]. A recent case report of sarcoptic mange in a potbelly pig described a failure of ivermectin treatment possibly due to resistant sarcoptic mites or expelling of the administered ivermectin by the animal; consequently, an isoxazoline derivate—afoxolaner—was successfully used at a dose of 2.6 mg/kg bodyweight as an oral treatment [15]. Herein, we report a case of demodicosis in a Kunekune pig from Austria, with data on the morphological and molecular characterization of the demodectic mites.

## Case report

### Herd description

The case animal lived in an educational farm in Lower Austria, Austria. The owner kept three Kunekune pigs in total, all 1 year of age, and five 3-year-old miniature pigs, as wells as goats, horses, guinea pigs, rabbits and chickens. The Kunekune and the other miniature pigs were kept together in an outdoor holding all year round, including a shelter with deep straw bedding for resting and sleeping. They were fed a commercial diet for miniature pigs; additionally, vegetables and fruits were offered every other day. Water was available ad libitum. The pig barn was separated from the enclosures of the other animal species. None of the pigs had ever been vaccinated. According to the owner, the pigs were routinely dewormed twice a year using injectable ivermectin.

### Anamnesis and clinical examination

In January 2021, the attending veterinarian of the case farm referred the affected 1-year-old female Kunekune pig to the University Clinic for Swine with a suspected diagnosis of bilateral entropion. The owner additionally reported that the patient had been smaller than its litter mates (runt) as a piglet. In August 2020, all pigs of the farm had suffered from sarcoptic mange which was successfully treated by injectable ivermectin. Thereafter, no further signs of pruritus or skin lesions were observed. The referred Kunekune pig was the only one in the herd suffering from a severely reduced visual field. Clinically, the herd veterinarian reported a moderate to severe thickening of the skin around both orbits, which also affected the eyelids. The owner stated that the animal was feeding and drinking normally and did not show any impairment of general behaviour or pruritus, with exception of a longer reaction time when targeting barriers such as fences, possibly because of the impaired vision. No treatments were attempted prior to the referral.

On referral, the patient had a body weight of 35.6 kg. All vital signs were within normal limits. Neither pruritus nor signs of rubbing could be observed. Plaque-like to nodular skin lesions up to 5 cm in diameter were present bilaterally on the periocular skin and on the lower and upper eyelids, resulting in a constriction of the palpebral opening (Additional file 1A). The skin in this area was alopecic, moderately hyperplastic with visibly enlarged noduli that were also present on the snout of the animal. A moderate number of multifocal skin-coloured papules and comedones was present in the axillar and inguinal regions (Additional file 1B and C).

### Differential diagnoses

Because miniature and Kunekune pigs of the case herd were affected by sarcoptic mange 6 months before, *Sarcoptes scabiei* var. *suis* was included in the list of differential diagnoses, even though the animal did not show any pruritus. Neoplastic lesions including swine papilloma were also considered, although the typical clinical presentation of exophytic, cauliflower-like hyperkeratotic lesions was lacking. Pyoderma was also included in the list of differential diagnoses.

### Surgical intervention

**Fig. 1:**
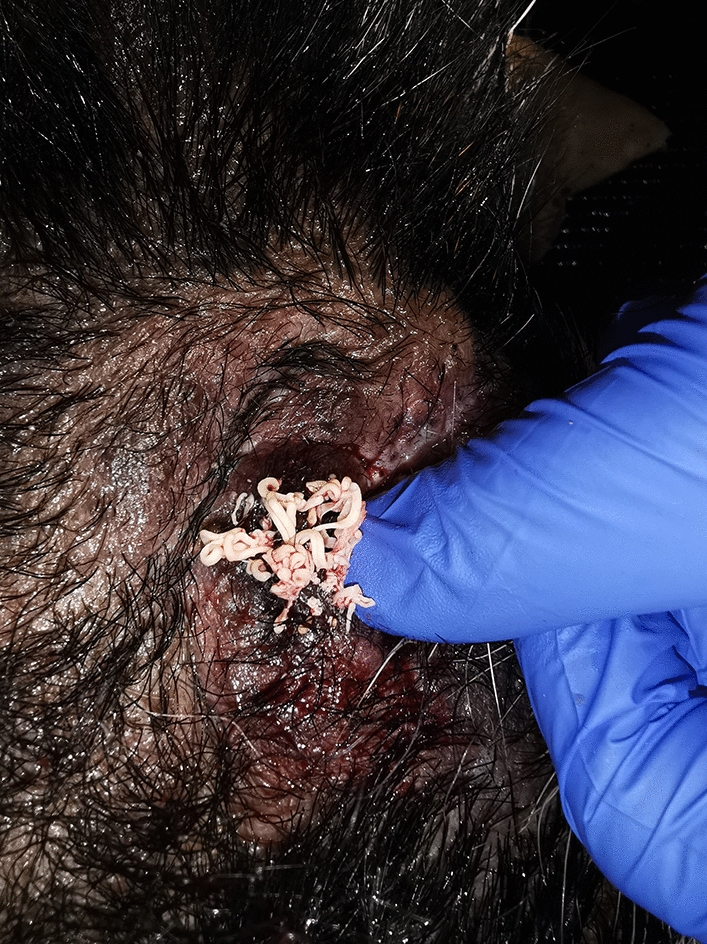
Right lower part of the periocular skin. Abundant whitish caseous material could be removed after squeezing by manual massage

To improve the range of vision of the animal as well as to obtain diagnostic samples, it was decided to surgically remove a part of the thickened periocular skin for histopathology as the next step in diagnostic workup, simultaneously increasing the visual field of the animal. Semilunar-shaped stripes of skin were removed from both upper eyelids in a modification of the Celsus-Hotz procedure for surgical correction of entropion [16]. Any painful intervention was done under general anaesthesia [intravenous administration of 10 mg/kg body weight ketamine (Ketamidor®, VetViva Richter GmbH, Wels, Austria)] and 2.5 mg/kg BW azaperon (Stresnil®, Elanco Animal Health, Bad Homburg, Germany). During the first general anaesthesia for surgical excision of hyperplastic periocular skin, soaking with warm and moist compresses followed by manual massage had been unproductive in removing caseous material from the massively enlarged skin pores in the periocular region. Therefore, in the second anaesthetic event the affected skin was repeatedly penetrated with a needle (18 gauge) for a better and more thorough removal of caseous material (Fig. 1 and Additional file 2). After surgery the pig was treated intramuscularly with 15 mg/kg bodyweight long-acting amoxicillin (Vetrimoxin L.A. 150 mg/ml, Ceva Sante Animale, Libourne, France) for 6 days to prevent bacterial secondary infections of the skin and with 0.4 mg/kg bodyweight meloxicam (Metacam 20 mg/ml, Boehringer Ingelheim Vetmedica GmbH, Ingelheim, Germany) for 3 days to reduce inflammation, swelling and pain. Caseous material obtained was stored at – 20 °C and fixed in 10% formalin and 70% EtOH for further investigations.

The wounds were closed using 3–0 monofilament and non-absorbable material by single-button sutures which were removed under general anaesthesia 7 days after surgery. The removed pieces of skin were directly transferred into 10% buffered formalin for fixation and further histopathological examination at the Institute of Pathology of the Vetmeduni Vienna.

### Histopathological investigations

**Fig. 2:**
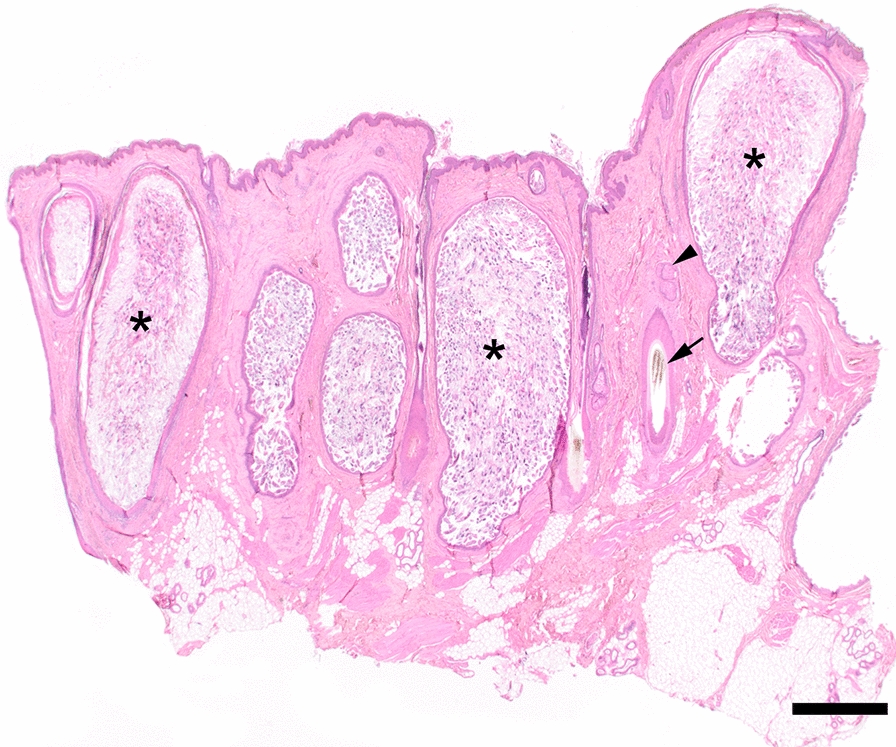
High-grade infestation of markedly hyperplastic hair follicles with *Demodex* mites (asterisk) as well as single unremarkable hair follicles (arrow) and sebaceous glands (arrowhead) (H&E staining, Scale bar = 1 mm)

**Fig. 3:**
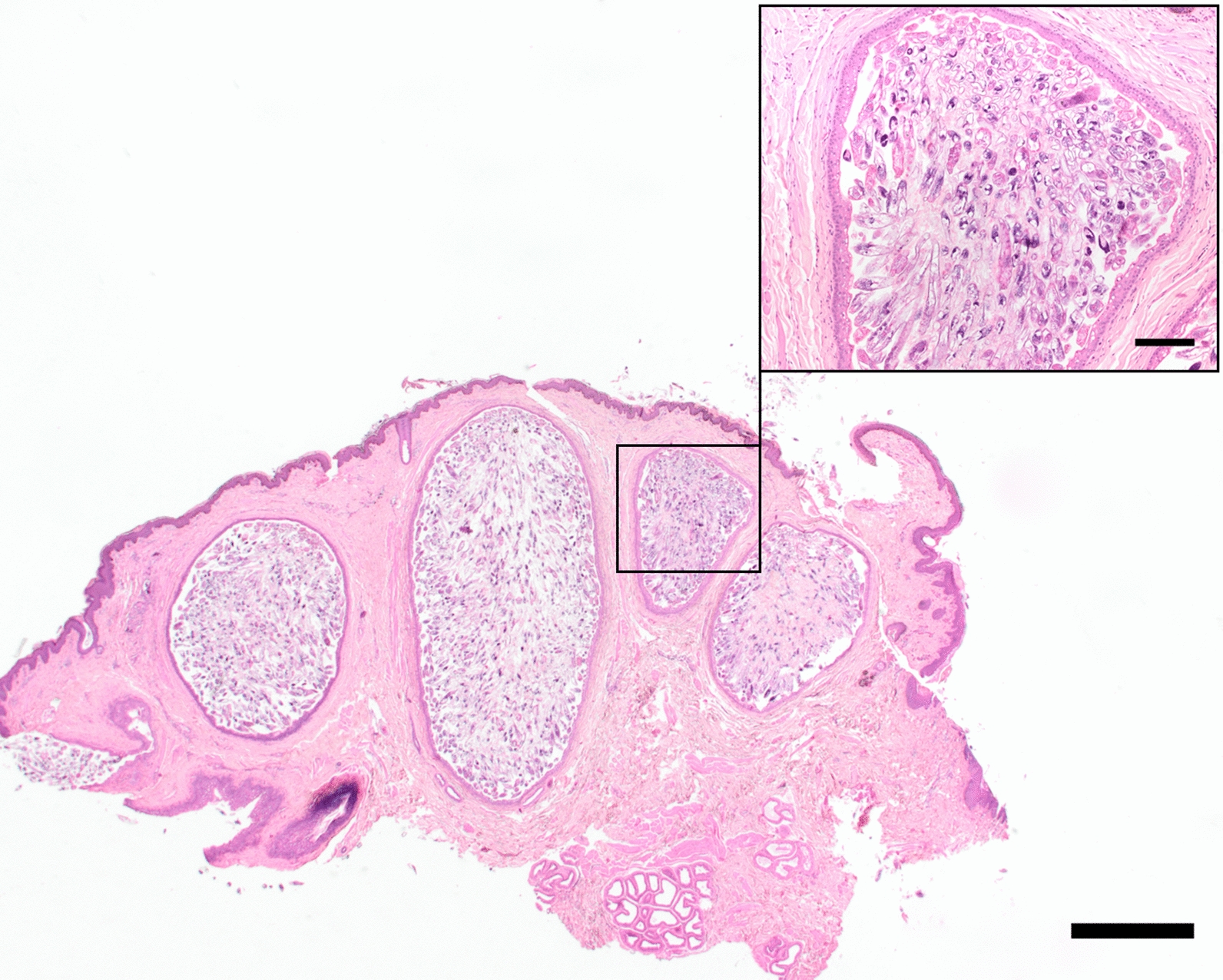
Skin sample with densely packed, highly hyperplastic hair follicles (H&E staining, Scale bar 1 mm); in the inset, masses of mites in different stages of development in a highly dilated hair follicle (H&E staining, Scale bar = 160 µm)

Macroscopically, the two surgically excised skin samples of the right and left upper eyelids, each measuring approximately 3 × 2 × 1 cm, showed a finely humped to nodular surface and an extremely sparse set of bristles. Histologically, the pattern was characterized by distinct, sac-like bulges of numerous hair follicles, whose lumina were filled with masses of parasites (Fig. 2). Eggs, larvae, nymphs and adult stages of *Demodex* mites were found in these highly hyperplastic hair follicles (Fig. 3). Sebaceous glands filled with mites were not detected in the available tissue samples. Moderate pyogranulomatous inflammation was evident around single, ruptured hair follicles, and low-grade, mostly perivascular, lymphoplasmacytic infiltrates were detectable in adjacent dermal tissue. Only sporadic regular hair follicles and sebaceous glands were present in the skin localizations examined. In addition, the sparse tubular glands were significantly dilated.

### Morphological and molecular characterization of porcine demodectic mites

**Fig. 4:**
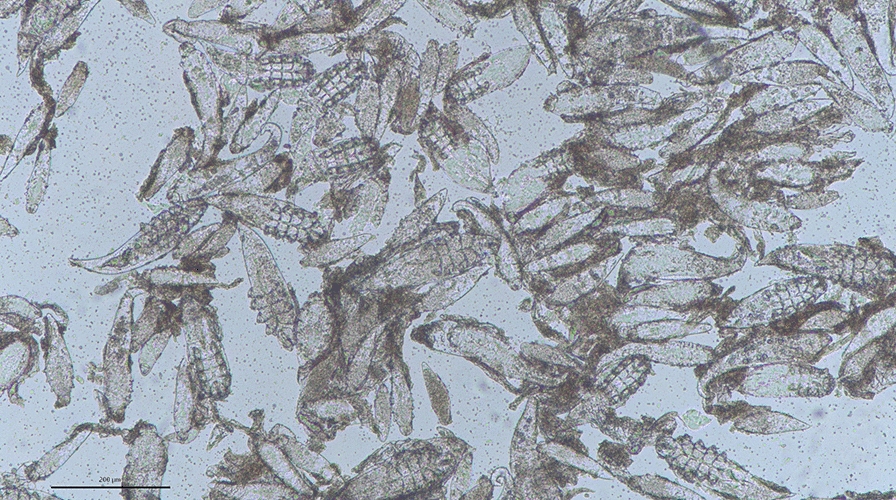
Light microscopy of caseous material fixed in 10% formalin. Different stages of *Demodex* mites. Scale bar = 200 µm

**Fig. 5:**
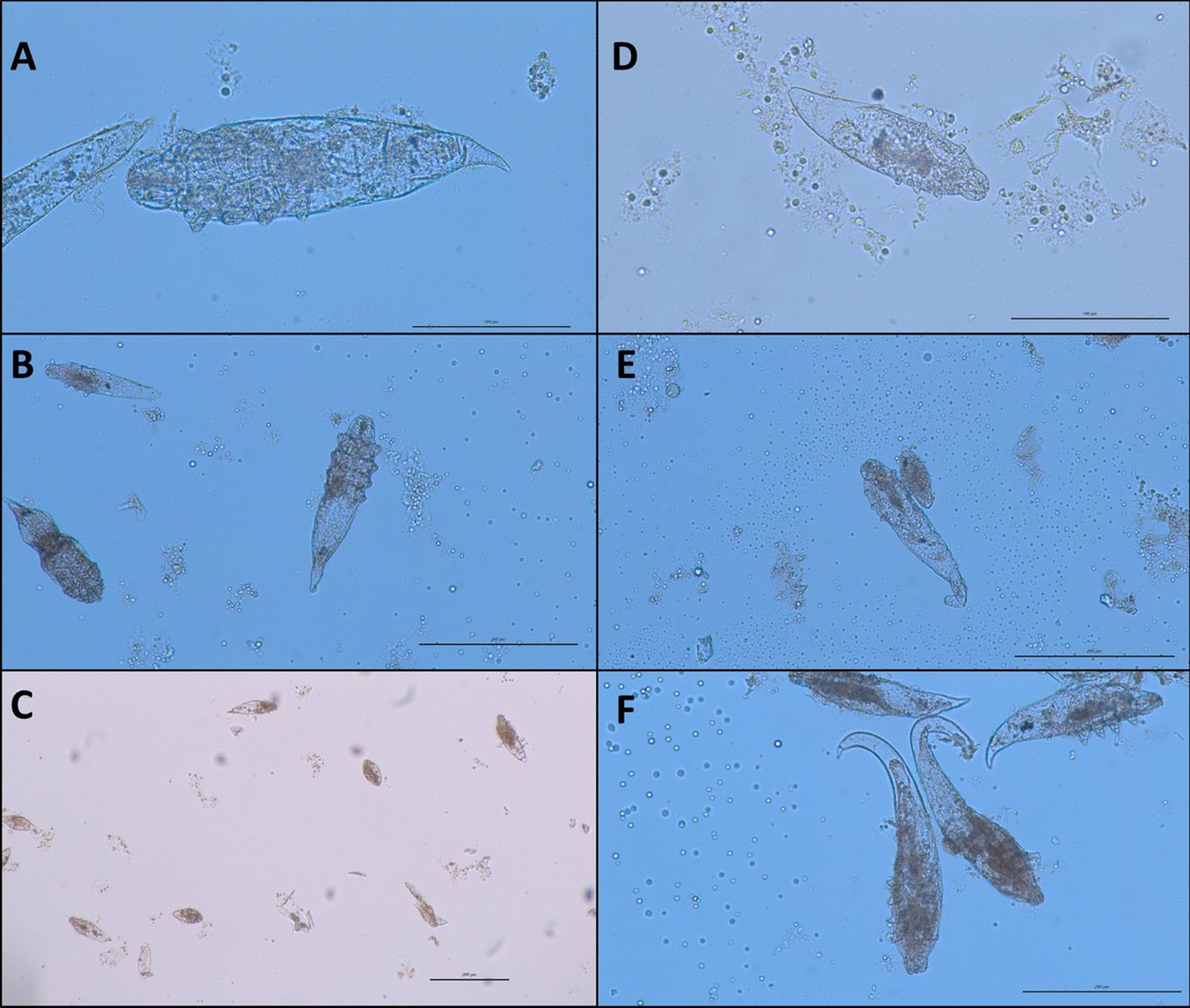
Light microscopic pictures of different developmental stages of *Demodex phylloides*. **A** = male adult mite, **B** = female adult mite, **C** = eggs, **D** = larva, **E** = protonymph, **F** = nymph. Scale bar = 200 µm

Samples of caseous material in 10% formalin and 70% EtOH were analysed native by light microscopy, and different developmental stages of *Demodex* mites were found (Fig. 4). Measurements were taken of all stages (Fig. 5) on a Nikon Eclipse Ci microscope with a Jenoptik Gryphax® Subra camera with the corresponding computer programme (Jenoptik AG, Germany). The shape, length, breadth and dimensions of the developmental stages were in the range of the data reported for *D. phylloides* in the literature [5, 17]. Also, the length and breadth of the gnathosoma, podosoma and opisthosoma from adult mites were in line (Additional file 3). The body is distinctly narrowing towards the end as described in the literature for this species [17].

**Fig. 6:**
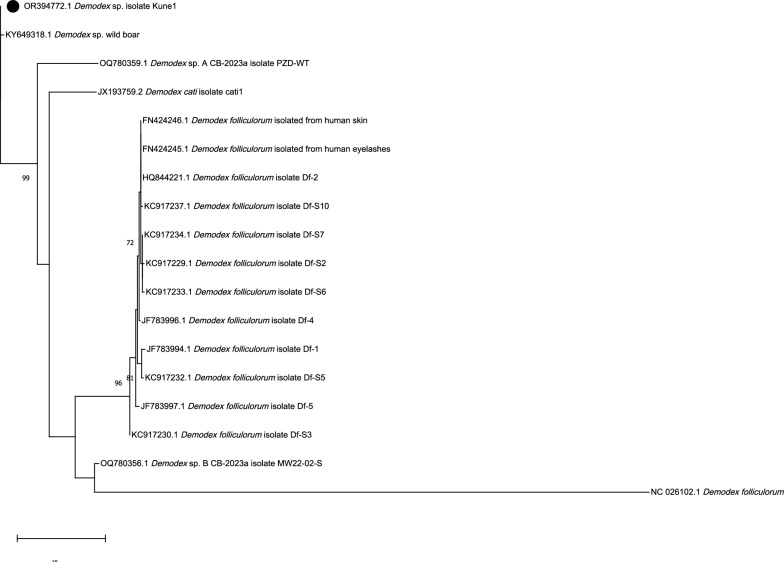
Phylogenetic analysis of the sequence. Numbers next to the nodes represent the bootstrap values; only bootstrap values > 70% are shown (1000 replicates). The scale bar represents a length corresponding to 0.2 nucleotide substitutions per site. Sequence obtained in this study is marked with a black dot. Other sequences were downloaded from GenBank

To further investigate the involved species, molecular analyses were performed to increase the availability of genetic data of porcine *Demodex* mites on the one hand and to possibly form the genetic base for *D. phylloides* on GenBank as the only described porcine *Demodex* species on the other. For this, DNA from frozen and stored caseous material was isolated using the QIAGEN DNeasy Blood and Tissue kit (QIAGEN, Hilden, Germany) and the High Pure PCR Template Preparation Kit (Roche, Vienna, Austria). Samples were incubated at 56 °C overnight, processed according to the manufacturer’s protocol and analysed by PCR targeting the 16S rDNA gene following the protocol from Zhao and Wu [18] on a Biometra Tone cycler (Analytik Jena, Germany). PCR products were analysed by electrophoresis on 2% agarose gels stained with Midori Green Advance DNA® stain (Nippon Genetics Europe, Düren, Germany). Both extraction methods returned identical results in PCR and sequence analysis. Amplicons were cycle sequenced using amplification primers (LGC Genomics GmbH, Germany); the obtained sequence was analysed using the BLAST function on NCBI (https://blast.ncbi.nlm.nih.gov/Blast.cgi) and uploaded to GenBank (accession no. OR394772). The highest similarity (98.68%) was obtained with *Demodex* sp. from a wild boar from Spain (accession no. KY649318). No explicit *D. phylloides* sequences were available in GenBank for comparison. A phylogenetic tree was generated (maximum likelihood method and Hasegawa_Kishino-Yano model [19] using MEGA version 11 [20] based on results of the alignment of the sequence of demodectic mites of this case with 18 published sequences on GenBank. Only sequences showing homology scores > 79% and a query cover of 100% were included in the phylogenetic tree (Fig. 6).

### Treatment and follow-up

Once the diagnosis was established, dermatologists at the Vetmeduni Vienna recommended an oral therapy with sarolaner (Simparica 80 mg chewable tablets for dogs > 20–40 kg, Zoetis Belgium SA, Louvain-la-Neuve, Belgium), an isoxazoline similar to the one used by Smith et al. for the treatment of sarcoptic mange [15]. The dose of 2.6 mg/kg sarolaner was also similar to the one used by this publication. The treatment was well tolerated, and no side effects were observed after oral administration of sarolaner. Eleven days after admission to the University of Veterinary Medicine Vienna, the affected Kunekune was discharged home.

The animal was visited after 1 month for re-evaluation of the skin lesions and for a second treatment with sarolaner. At that time point, the skin lesions were still present but in regression. The palpebral fissures were not constricted anymore, and the patient's behaviour was consistent with a larger visual field. The owner declined a follow-up skin scraping for detection of *Demodex* mites. During this visit, the remaining pigs of that farm were clinically investigated, and none of them showed signs indicative of clinical demodicosis. After 8 months the skin lesions of the animal were in remission and thickened skin parts were slightly visible, probably because of cicatrisation of the hair follicles. The owner reported that the animal was not impaired visually anymore (Additional file 1D).

## Discussion

In the present case report of porcine demodicosis, a 1-year-old female Kunekune pig presented clinically with multifocal papular to nodular skin lesions, which severely constricted the eyelid opening and impaired vision of the affected animal. The diagnosis was reached by histopathologic examination of skin biopsies and the parasitological identification of responsible mites. Based on morphometrics, *D. phylloides* was the most likely involved species. However, the lack of reference samples in GenBank prevented a molecular confirmation at the species level. Treatment with sarolaner resulted in almost complete clinical resolution, as 8 months after the treatment only a slight thickening of the periorbital skin was present. Incontact animals were not affected.

In many animal species as well as in humans, presence of *Demodex* mites in clinically normal skin has been described. Clinical disease with skin lesions develops in presence of factors such as immune dysregulation or immunosuppression [21]. Marginal blepharitis in humans is confined to the meibomian glands at the eyelid margin and therefore much less severe than the extended skin lesions of the pig in this case report.

In humans, one of the possible presentations of demodicosis is blepharitis and ocular demodicosis. Although one older study found all human participants in that study > 70 years old to have *Demodex* mites present in the hair follicles of their eye lashes without clinical disease, the presence of factors such as immunosuppression, diabetes mellitus, vasodilatation factors and/or sebaceous gland hyperplasia may trigger the proliferation of *Demodex* spp. [22–24].

Only scarce information regarding subclinical *Demodex* mite infestation and porcine demodicosis can be found in the literature [1–3, 6, 10, 11]. Historically, demodicosis was thought to be more economically relevant than it is now, as swine skin was a not negligible source for leather production, and demodicosis was an important cause of reduced leather quality [5]. Demodicosis in conventional pigs may also have become rare because of the frequent use of macrocyclic lactones for the control of gastrointestinal nematodes and ectoparasites such as sarcoptic mange. In the last 20 years, only two papers on demodicosis in pigs, both from Brazil, have been published [7, 8]. Factors such as stress, poor husbandry and biosecurity conditions as well as inadequate nutrition have been implicated previously [7, 25]. Regarding demodicosis in miniature pigs or in pig breeds kept as companion animals (e.g. Kunekune), no literature exists. No reports on the pathogenesis of porcine demodicosis are available, but one may speculate that, as suggested for demodicosis in other hosts, *D. phylloides* can reproduce freely in a host with immune dysregulation or immunosuppression, resulting in high mite numbers and consequently skin lesions [26]. In contrast to previous publications, the Kunekune pig presented to Vetmeduni Vienna was in good general health, showing no obvious signs of immunosuppression at the examination and was kept in good husbandry conditions. It was the only animal in the herd with skin lesions, which is also in contrast with previous reports. Based on anamnestic information, the patient was a runt as a piglet, making immune alterations a possibility.

According to the owner, no fights were observed between the patient and other pigs, but she stated that during feeding and social interaction with her as animal caretaker, the case animal was always the last one, suggesting a rather lower social rank in the herd. This observation allows for speculation about stress as an aetiological factor.

The molecular typing of *Demodex* sp. in the case animal clearly showed a close relation with a published sequence originating from demodectic mites isolated from a wild boar as host species (98.68%). The next closest related *Demodex* species was *Demodex cati* from a cat from the USA with 84.64% identity (JX193759), *Demodex* sp. on a house mouse (*Mus musculus*; OQ780356) from Australia with 84.64% and *Demodex folliculorum* (KC917234) collected on a human in China with 80.46% identity. Recent studies investigating the prevalence of *Demodex* of porcine hosts do not exist; hence, it just can be speculated about their prevalence and clinical relevance. For the future, systematic molecular investigations have to be done on *Demodex* isolated from porcine hosts to generate a robust genetic data base of *Demodex* sequences on the one hand and on the other hand to finally examine if there is only one species of demodectic mites affecting pigs. Cats are known to be affected by more than one *Demodex* species, making this a reasonable suspicion in pigs [27, 28]. More sequence data would allow to determine whether there are always the same or different genotypes detectable. As morphometrics of different developmental stages of demodectic mites of the Kunekune met the discrimination criteria of *D. phylloides* in already published studies, we probably generated the first confirmed genetic base of *D. phylloides*.

An impactful textbook on swine medicine states that there are no reports of successful treatment of porcine demodicosis [4, 29]. However, in addition to the present case report, two other case reports demonstrated successful treatment using in-feed ivermectin at a dose of 0.45 g/kg of feed for seven days. Absence of *Demodex* mites 15 days after treatment was confirmed by examinations of skin scrapings [7, 8].

Only macrocyclic lactones (ivermectin, doramectin) are currently registered for the treatment of porcine demodicosis. These are described to be efficacious for the treatment of demodicosis in pigs for pork production [7, 8]. However, in a recent case report lack of efficacy of ivermectin against sarcoptic mites in a potbellied pig was described and the authors decided to use afoxolaner as an alternative to treat sarcoptic mange effectively [15]. As the affected animal in the present case was registered as a companion animal, not assigned for meat production, it was decided to look for more effective alternatives for the treatment of demodicosis. Fluralaner is registered for control of the poultry red mite in chicken [30], but currently no isoxazoline derivates are registered for use in pigs. It was decided to use sarolaner off label for treating demodicosis in this animal patient. So far, treatment has been well tolerated without the observance of any adverse effects. Re-evaluation of the clinical status of that pig 1 month after the start of the treatment resulted in an almost complete clinical recovery and a remission of the skin alterations. This was considered a therapeutic success. The veterinary products administered to the patient were almost all not registered for their intended use in this case. Thus, off-label use of medical products always must consider legal requirements in the country in which corresponding patients are treated. However, we build the base for an off-label treatment scheme of future similar cases of *Demodex* infestations in pigs kept as companion animals.

Prognosis of demodicosis in pigs does not seem to be as poor as described in the literature. At least in this case report it was possible to successfully treat the affected animal. As isoxazolines are highly lipophilic [31] these substances accumulate in sebaceous material, which is also ingested by demodectic mites. Based on the results of the animal reported in this case report, it is assumed that treatment of pigs with isoxazolines is safe and highly efficacious with a good prognosis.

## Conclusions

Clinical demodicosis in pigs is an uncommon diagnosis and should be considered in cases with multifocal papular to nodular skin lesions affecting predominantly the facial region. While of low relevance in commercial pigs, miniature pigs and pigs kept as companion animals can be affected by this disease. As this group of pig population is gaining popularity, repurposing of drugs registered for pigs kept as companion animals can be considered following legal considerations. In cases of periocular alterations of the skin, demodectic mange should be considered as a differential diagnosis.

### Supplementary Information

**Additional file 1.** Left orbital and periocular region of the affected Kunekune pig with severe hyperplastic skin (**A**). Skin presented with nodules and comedones in the axillar (**B**) and inguinal (**C**) region. Case animal 1 month after surgical and antiparasitic intervention. The eyelids are opened and the eye clearly can be seen (**D**). Thickened skin around the orbits still is present but without reducing the visual field or vision respectively.

**Additional file 2.** Demonstration of the process of needling for efficient removal of caseous material from affected skin parts around the orbits.

**Additional file 3.** Morphometric data on different developmental stages of *Demodex* mites.

## Data Availability

All data generated or analysed during this case report are included in this published article or are available as supplementary data.

## References

[1] Csokor J (1879). Über Haarsackmilben und eine neue Varietät derselben bei Schweinen, *Demodex phylloides*. Verh Zool Bot Ges Österr..

[2] Wright RR (1883). On *Demodex phylloides*, (Csokor), in the skin of Canadian swine. Proc R Canad Inst (Toronto)..

[3] Rieck oV. *Demodex phylloides*. Bericht über das Veterinärwesen im Königreiche Sachsen. 1901; 45:53–4

[4] Brewer MT, Greve JH, Zimmerman JJ, Karriker LA, Ramirez A, Schwartz KJ, Stevenson GW, Zhang J (2019). External parasites. Diseases of Swine.

[5] Rehbein S, Visser M, Oertel H. Demodikose beim Schwein—Klinisches Bild, Milbenpopulation und Schäden am Leder. Tierarztl Umsch. 1999:38–46.

[6] Rapp J, Koch F. Demodikose beim Schwein. Vet med Nachrichten. 1979:67–9.

[7] Bersano JG, Mendes MC, Duarte FC, Del Fava C, de Oliveira SM, Filha ES (2016). *Demodex phylloides* infection in swine reared in a peri-urban family farm located on the outskirts of the Metropolitan Region of São Paulo. Brazil Vet Parasitol.

[8] Santarém VA, Farias MR, Tostes RA (2005). Demodectic mange in fattening pigs in São Paulo. Brazil Vet Parasitol.

[9] Fryderyk S, Izdebska JN (2001). *Demodex phylloides* (Acari, *Demodecidae*) as a specific parasite of *Sus scrofa* (Mammalia, Artiodactyla). Wiad Parazytol.

[10] Nemeséri L, Széky A (1966). Demodicosis of swine. Acta Vet Acad Sci Hung.

[11] Nutting WB (1976). Hair follicle mites (*Demodex* spp.) of medical and veterinary concern. Cornell Vet.

[12] Baars G, Müller J. Ein Fall von *Demodex*-(Acarus-) Räude beim Schwein in der Lebensmittelkontrolle. Z Fleisch Milchhyg. 1930:267–9.

[13] Scott DW (1988). Large animal dermatology.

[14] Scott DW (2018). Color atlas of farm animal dermatology.

[15] Smith JS, Berger DJ, Hoff SE, Jesudoss Chelladurai JRJ, Martin KA, Brewer MT (2020). Afoxolaner as a treatment for a novel *Sarcoptes scabiei* infestation in a juvenile potbelly pig. Front Vet Sci.

[16] Hotz C (1979). Operation for entropion. Arch Ophthal.

[17] Izdebska JN (2009). Selected aspects of adaptations to the parasitism of hair follicle mites (Acari: Demodecidae) from hoofed mammals. Eur Bison Conserv Newslett.

[18] Zhao Y-E, Wu L-P (2012). Phylogenetic relationships in *Demodex* mites (Acari: Demodicidae) based on mitochondrial 16S rDNA partial sequences. Parasitol Res.

[19] Hasegawa M, Kishino H, Yano T (1985). Dating the human-ape split by a molecular clock of mitochondrial DNA. J Mol Evol.

[20] Tamura K, Stecher G, Kumar S (2021). MEGA 11: molecular evolutionary genetics analysis version 11. Mol Biol Evol.

[21] Izdebska JN, Rolbiecki L (2020). The biodiversity of demodecid mites (Acariformes: Prostigmata), specific parasites of mammals with a global checklist and a new finding for *Demodex sciurinus*. Diversity.

[22] Czepita D, Kuźna-Grygiel W, Czepita M, Grobelny A (2007). *Demodex folliculorum* and *Demodex brevis* as a cause of chronic marginal blepharitis. Ann Acad Med Stetin.

[23] Post CF, Juhlin E (1963). *Demodex folliculorum* and blepharitis. Arch Dermatol.

[24] Forton FMN (2020). The pathogenic role of *Demodex* mites in rosacea: a potential therapeutic target already in erythematotelangiectatic rosacea?. Dermatol Ther (Heidelb).

[25] Torrison J, Ranald Cameron R, Zimmerman JJ, Kar-riker LA, Ramirez A, Schwartz KJ, Stevenson GW, Zhang J (2019). Integumentary system. Diseases of swine.

[26] Gazi U, Taylan-Ozkan A, Mumcuoglu KY (2019). Immune mechanisms in human and canine demodicosis: a review. Parasite Immunol.

[27] Silbermayr K, Horvath-Ungerboeck C, Eigner B, Joachim A, Ferrer L (2015). Phylogenetic relationships and new genetic tools for the detection and discrimination of the three feline *Demodex* mites. Parasitol Res.

[28] Ferreira D, Sastre N, Ravera I, Altet L, Francino O, Bardagí M (2015). Identification of a third feline *Demodex* species through partial sequencing of the 16S rDNA and frequency of *Demodex* species in 74 cats using a PCR assay. Vet Dermatol.

[29] Zimmerman JJ, Karriker LA, Ramirez A, Schwartz KJ, Stevenson GW, Zhang J (2019). Diseases of Swine.

[30] European Medicines Agency. Exzolt. 2023. https://www.ema.europa.eu/en/medicines/veterinary/EPAR/exzolt. Accessed 24 Aug 2023.

[31] Gonçalves IL, Machado das Neves G, Porto Kagami L, Eifler-Lima VL, Merlo AA. Discovery, development, chemical diversity and design of isoxazoline-based insecticides. Bioorg Med Chem. 2021;30:115934. 10.1016/j.bmc.2020.115934 33360575

