# Supplementary figures and images for: Demodicosis in a Kunekune pig and molecular characterisation of porcine demodectic mites involved: a case report
Source: Parasit Vectors. 2024 Jan 24;17:32. doi: 10.1186/s13071-023-06101-8 (PMC10807167; doi:10.1186/s13071-023-06101-8)

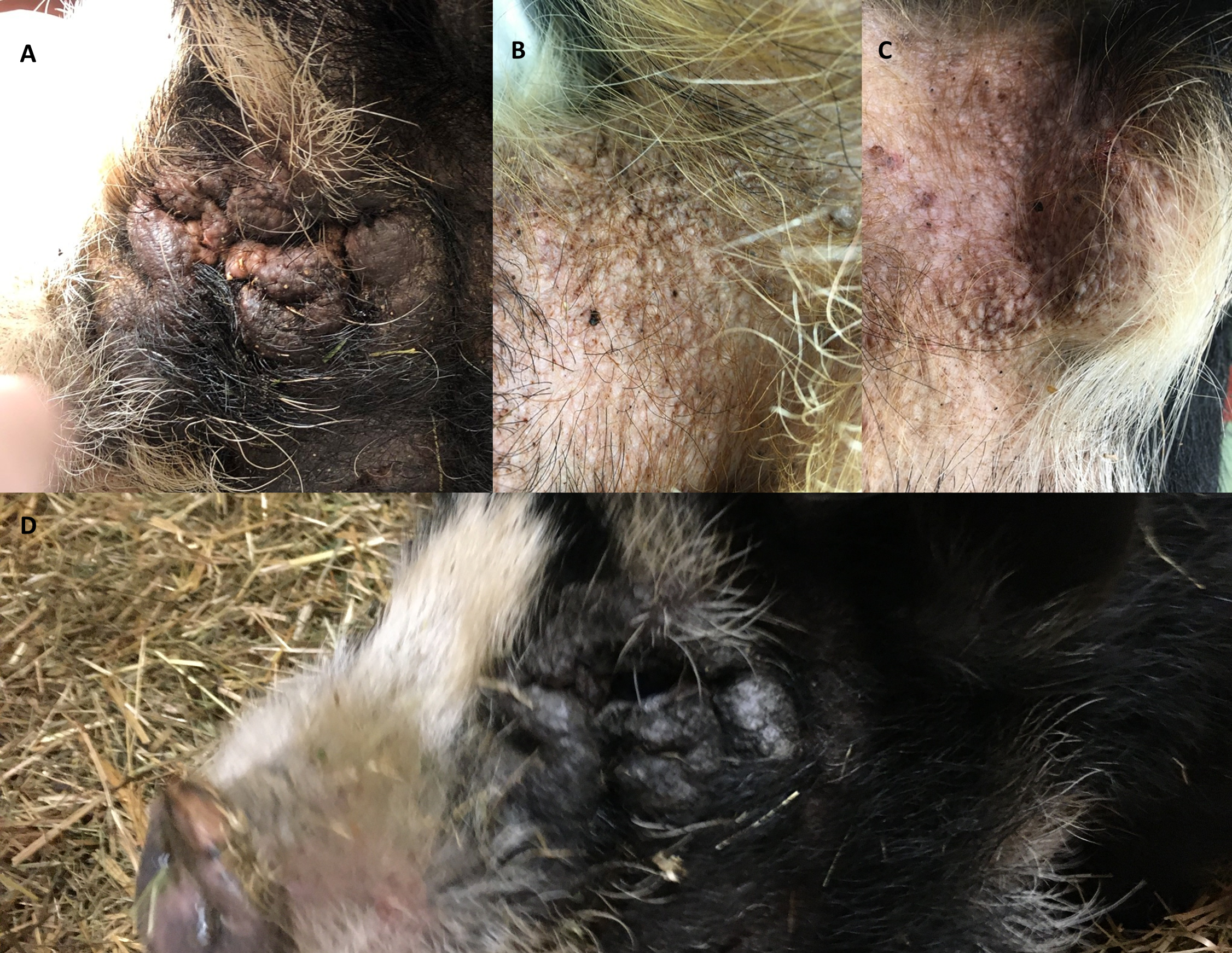

Supplement: Supplementary file 1 — Additional file 1. Left orbital and periocular region of the affected Kunekune pig with severe hyperplastic skin (A). Skin presented with nodules and comedones in the axillar (B) and inguinal (C) region. Case animal 1 month after surgical and antiparasitic intervention. The eyelids are opened and the eye clearly can be seen (D). Thickened skin around the orbits still is present but without reducing the visual field or vision respectively. [file 13071_2023_6101_MOESM1_ESM.tif]
